# Draft Genome Sequences of *Dickeya* Species Associated with Soft Rot Diseases in Pineapple (Ananas comosus) and Banana (*Musa* spp.)

**DOI:** 10.1128/mra.00716-22

**Published:** 2022-10-05

**Authors:** Anthony J. Young, Henry W. G. Birt, Niloofar Vaghefi, Robert D. Hoelzle, Paul G. Dennis

**Affiliations:** a School of Agriculture and Food Sciences, The University of Queensland, Gatton, Queensland, Australia; b School of Earth and Environmental Sciences, The University of Queensland, Brisbane, Queensland, Australia; c School of Agriculture and Food, Faculty of Veterinary and Agricultural Sciences, University of Melbourne, Parkville, Victoria, Australia; SIPBS, University of Strathclyde

## Abstract

*Dickeya* species cause soft rots on many commercial crops. Here, we present the draft genomes of Dickeya oryzae (BRIP 64262) and Dickeya zeae (BRIP 64263) isolates causing soft rot on banana (*Musa* spp.) and pineapple (Ananas comosus) plants, respectively. This expands the range of available genomes from plant-pathogenic *Dickeya* species.

## ANNOUNCEMENT

*Dickeya* species cause soft rots on a wide range of plants, including potato, maize, rice, pineapple, and banana (1–5). While draft and complete genome sequences are available for Dickeya zeae and Dickeya oryzae strains from North America, Europe, and Asia (6–9), none has been reported from Australia. Here, we present draft genome sequences for two isolates, D. oryzae BRIP 64262, sourced from banana (*Musa* spp.), and D. zeae BRIP 64263, sourced from pineapple (Ananas comosus).

Banana samples exhibiting corm rot were collected from a field at South Johnstone, Queensland, Australia (17°36′19.0″S, 145°59′54.6″E). Corms were split to reveal infected tissue, and streaks onto Kelman’s medium were made from the margin of infection. Bacteria were isolated in the same way from the necrotic margins of symptomatic pineapple plants (1). Plates were incubated aerobically at 28°C for 3 days until individual colonies appeared, which were then purified by three successive streaks from single colonies. DNA was extracted using the Isolate II plant DNA extraction kit (Bioline). Briefly, ~250 mg of biomass was transferred from solid medium into a 2-mL screw-cap tube containing extraction buffer and a 4-mm-diameter steel ball and then was processed in a FastPrep bead beater for 40 s at a setting of 4.5 m s^−1^ before purification according to the manufacturer’s instructions. DNA libraries were prepared using the Nextera XT DNA library preparation kit (Illumina, Inc., San Diego, CA, USA) and sequenced on the MiSeq platform with 150-bp paired-end reads (Illumina, Inc.), generating 296 Mbp and 333 Mbp of data from 2.00 million (BRIP 64262) and 2.25 million (BRIP 64263) read pairs, respectively.

Raw reads were trimmed and quality filtered with Trimmomatic v0.36 (10) using the following settings: ILLUMINACLIP:NexteraPE-PE.fa:2:30:10, TRAILING:10, SLIDINGWINDOW:4:15, and MINLEN:75. Paired-end trimmed reads were *de novo* assembled using SPAdes v3.14.1 (11) in isolate mode. The assembled contigs were checked for completeness and contamination using CheckM v1.1.2 (12) and assigned taxonomy using GTDB-Tk v1.3.0 and the Genome Taxonomy Database (GTDB) 07-RS207 release (13). The NCBI Prokaryotic Genome Annotation Pipeline (PGAP) v6.1 was used to identify genes (14). Average nucleotide identity (ANI) was assessed with FastANI v1.3 (15). Default parameters were used unless otherwise specified.

The BRIP 64262 and BRIP 64263 draft genomes were of similar sizes (4.7 and 4.8 Mbp, respectively), compared to neighboring species, according to phylogenetic analysis, and were alike in composition, with 94.7% ANI. BRIP 64262 had 97.2% ANI to the D. oryzae type strain (GenBank assembly accession number GCF_009372235.1), while BRIP 64263 had 96.3% ANI to the D. zeae type strain (GenBank assembly accession number GCF_002887555.1). The BRIP 64262 and BRIP 64263 draft genomes were assembled into 191 and 173 contigs, with *N*_50_ values of 402 and 194 kbp, respectively, and both had GC contents of 58%. According to CheckM, the draft genomes of BRIP 64262 and BRIP 64263 were both 99.97% complete, with 0.61% and 1.00% contamination, respectively. Both isolates had 3 copies of the 16S rRNA gene, while PGAP annotated 4,234 genes for BRIP 64262 and 4,293 genes for BRIP 64263, similar to comparable genomes of D. zeae (GenBank assembly accession number GCF_002887555.1) (4,216 genes) and D. oryzae (GenBank assembly accession number GCF_009372235.1) (4,031 genes).

### Data availability.

The data from this project have been deposited under NCBI BioProject accession number PRJNA737524. The raw sequencing data have been deposited in the Sequence Read Archive (SRA) under accession numbers SRR14876326 (BRIP 64262) and SRR14876325 (BRIP 64263), and the draft genome assemblies have been deposited in GenBank (BRIP 64262, BioSample accession number SAMN19735995; BRIP 64263, BioSample accession number SAMN19735996). The versions described in this paper are the first versions (BioSample accession numbers SAMN19735995 and SAMN19735996). The annotated genomes are available in GenBank under the following accession numbers: BRIP 64262, JAMXSN000000000; BRIP 64263, JAMXSO000000000.
